# Characteristics and accurate identification of *Pantoea dispersa* with a case of spontaneous rupture of hepatocellular carcinoma in China: A case report

**DOI:** 10.1097/MD.0000000000028541

**Published:** 2022-01-14

**Authors:** Yang Yang, Haitao Hu, Chenglin Zhou, Wenyun Zhang, Yang Yu, Qingyi Liu, Taohong Lu, Qingfang Zhang

**Affiliations:** Clinical Laboratory Center, Jiangsu Taizhou People's Hospital, Taizhou, Jiangsu, PR China.

**Keywords:** accurate identification, characteristics, *Pantoea dispersa*

## Abstract

**Introduction:**

*Pantoea dispersa* belongs to the genus *Pantoea*, which is isolated from *Enterobacteriaceae*. It has been reported to cause some kinds of infections, but there are few detailed studies on it, especially its characteristics and identification methods, which has caused a lot of trouble in clinical work.

**Patient concerns:**

A 51-year-old Chinese man was admitted to our hospital with a 7-hour history of progressive abdominal pain. He was previously diagnosed with liver cirrhosis secondary to chronic hepatitis B infection and hepatocellular carcinoma. An emergency hepatic artery embolization for hemostasis was performed under local anesthesia. Forty-eight hours later, the patient presented sudden onset of high fever up to 39.0 °C and chill.

**Diagnosis:**

Morphological and phenotypic profiles were performed for preliminary identification for *P dispersa*. The biochemical features were obtained by VITEK 2 Test Kit. Matrix-assisted laser desorption/ionization time-of-flight mass spectrometry analysis and 16S ribosomal RNA sequencing were performed to accurately identify *P dispersa*.

**Intervention:**

Antibiotic therapy of intravenous ceftazidime was started empirically. The antibiotic treatment was switched to intravenous cefepime at the same time because of suspected ceftazidime treatment failure and microbiological sensitivity.

**Outcomes:**

The patient remained afebrile, and the second blood culture results were negative. Chest X-ray was normal as well. In order to control the progression of the hepatic lesion, transarterial chemoembolization was performed under local anesthesia. After completion of 14 days of antibiotic treatment, the patient was discharged with no signs of recurrence.

**Conclusion:**

*P dispersa*, a gram-negative bacterium rod, were facultative anaerobic, which displayed yellow pigmentation, round, raised, smooth on culture plates. Conventional analysis was difficult to complete its identification. With biochemical tests, matrix-assisted laser desorption/ionization time-of-flight mass spectrometry analysis and 16S ribosomal RNA sequencing, *P dispersa* can be accurately identified. It will help physicians understand the related clinical manifestations and make timely and effective treatment for patients.

## 1 Introduction

The pathogenicity of *Enterobacteriaceae* in human diseases has been more and more recognized in past decades, and with the increase of bacterial resistance, the treatment for *Enterobacteriaceae* become less effective. Genus *Pantoea* is isolated from *Enterobacteriaceae* in 1989. The genus *Pantoea* is classified into 20 different species named *Pantoea eucalyptii*, *Pantoea agglomerans*, *Pantoea vagans*, *Pantoea conspicua*, *Pantoea deleyi*, *Pantoea anthophila*, *Pantoea brenneri*, *Pantoea ananatis*, *Pantoea allii*, *Pantoea stewartii*, *Pantoea cypripedii*, *Pantoea calida*, *Pantoea gavinae*, *Pantoea dispersa*, *Pantoea septica*, *Pantoea wallisii*, *Pantoea eucrina*, *Pantoea rodasii*, *Pantoea rwandensis*, and *Pectobacterium carotovorum*. The representative strain is *Pantoea agglomerans*.^[1]^ This kind of bacteria inhabits plants, soil and water, and rarely causes human infections.^[2]^*Pantoea dispersa* (*P dispersa*) is a kind of the genus *Pantoea*, which is often isolated from plants, closely related to *Erwinia*. Although this strain has been isolated from blood culture samples of a few patients abroad, there are still few reports in the literature of clinically significant infections involving this gram-negative rod.^[3,4]^ Here, we concluded the characteristics and accurate identification of *P dispersa* with the first report of *P dispersa* isolated from an immunocompromised man with spontaneous rupture of hepatocellular carcinoma in China.

## 2 Materials and methods

### 2.1 Blood cultures

Two sets of blood cultures were performed on the patient. The blood cultures were collected at the clinical ward and then transferred to our laboratory according to standard guidelines. The following blood culture bottles were used: BacT/Alert FA, and BacT/Alert FN blood culture bottles (BCBs) (bioMérieux). The bottles were incubated in the BacT/Alert 3D automated blood culture system (bioMérieux) until positivity or for a maximum incubation time of 5 days.

### 2.2 Short-term culture

All positive BCBs (irrespective of BCB type, whether anaerobic or aerobic) that are Gram stained, would have an associated short-term culture performed on different agars. The short-term culture was done by transferring several drops of broth with an inoculation needle onto different agar plates and streaked with bacteriological loops (10 μL). Incubation was performed at 37 °C in a 5% CO_2_ environment or put it in an anaerobic bag for 24, 48 hours respectively. Growth on the media would be checked for between 2 and 8 hours.

### 2.3 Bacterial isolation and identification

*VITEK test.* For the VITEK-2 platform, a bacterial suspension using 0.45% sodium chloride solution (bioMérieux, France) was adjusted to an optical density of 0.5 to 0.63 McFarland units. The VITEK-2 GN and VITEK-2 AST-N335/XN04 test card (bioMérieux, France) was inoculated with the bacterial suspension following the manufacturer's instructions. The cards were loaded into the VITEK-2 system and results were automatically reported by VITEK-2 Software release 8.01.

*MALDI-TOF MS*. Matrix-assisted laser desorption/ionization time-of-flight mass spectrometry (MALDI-TOF MS) was performed directly from bacterial material on plates. A very thin layer from visible bacterial growth was scraped with a 1 mL bacterial loop and smeared on 1 spot of a 96-spot stainless steel target plate (Bruker Daltonik). On-plate extraction was performed by overlaying each spot with 1 mL 70% formic acid solution (Fluka) and then allowing the samples to air dry for 2 minutes. One microlitre of CHCA matrix (a-cyano-4- hydroxycinnamic acid) in 50% acetonitrile was then applied on each sample and air-dried before analysis with MALDI-TOF MS. The Myla version 3.0 software and library (version 4613; Bruker Daltonik) was used for spectra analysis. Following the manufacturer's instructions, scores of i1.7 were considered as low-confidence identification but interpreted as a successful identification if confirmed by conventional methods obtained from overnight cultures. Scores of v1.7 were considered as failed identification.

*DNA extraction and PCR amplification of 16S rRNA gene.* The bacterial isolates were identified by sequence analysis of the 16S ribosomal RNA (rRNA) gene. Genomic DNA was isolated from overnight cultures using Ezup Column Bacteria Genomic DNA Purification Kit (Sanggon Biotechnologies, Shanghai, China) according to the manufacturer's instructions. The 16S rRNA was amplified by polymerase chain reaction (PCR) using the universal primers 27F (5′-AGTTTGATCMTGGCTCAG-3′) and 1492R (5′-GGTTACCTTGTTACGACTT-3′), which were used to amplify approximately 1.5-kbp segment of the 16S rRNA gene. The PCR reaction system was 25 μL according to the instructions in the kit (MBI, Ferment) and thermal cycling was performed in automatic PCR instrument (Applied Biosystems, Thermo Fisher Scientific) (Table S1, Table S2, Supplemental Digital Content). Amplification products were checked by 1.5% (w/v) agarose gel electrophoresis. PCR products were then sequenced (Sanggon Biotechnologies, Shanghai, China). The sequences were compared against the sequences in the National Centre for Biotechnology Information (NCBI) nonredundant database by using the BLASTn program (https://www.ncbi.nlm.nih.gov/).

### 2.4 Phylogenetic analysis

Multiple alignments of nucleotide gene sequences were created using the program MEGA 7.1.0 software (Mega Limited, Auckland, New Zealand). The neighbor-joining method with p-distance method was used to construct phylogenetic trees. The robustness of individual branches was estimated by bootstrapping with 1000 replications.

### 2.5 Antimicrobial-susceptibility testing

Antimicrobial-susceptibility test was performed on the isolated and cultured strains according to Clinical and Laboratory Standards Institute criteria for Enterobacteriaceae using the Vitek-2 8.0 automated microbiology system (bioMérieux, France). Etest was used for antimicrobial-susceptibility test throughout the study period and the minimum inhibitory concentrations were defined after 6 hours incubation.

### 2.6 Case presentation

**Figure 1:**
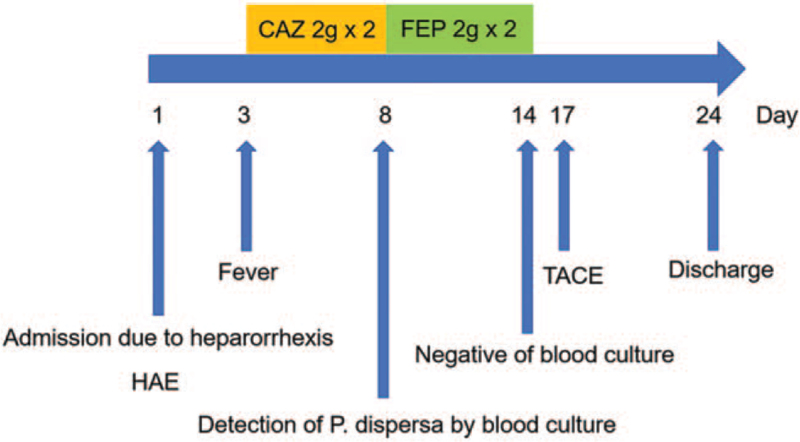
The clinical course of this case. CAZ = ceftazidime, HAE = hepatic artery embolization, TACE = transarterial chemoembolization.

A 51-year-old Chinese man was admitted to hospital on November 7, 2020 with a 7-hour history of progressive abdominal pain (Fig. 1). He was previously diagnosed with liver cirrhosis secondary to chronic hepatitis B infection and hepatocellular carcinoma. Based on the patient's symptoms, signs and computed tomography (CT) findings, he was diagnosed as the spontaneous rupture of hepatocellular carcinoma. An emergency hepatic artery embolization for hemostasis was performed under local anesthesia without any immediate complications.

Forty-eight hours later, the patient presented sudden onset of high fever up to 39.0 °C and chill. The laboratory test showed in Table 1. Antibiotic therapy of intravenous ceftazidime (1 g every 8 hours) was started empirically at the same time. He was afebrile for only 1 day following the antibiotic therapy, after which his body temperature rose to 38 °C with chill again. Therefore, 2 sets of blood cultures were obtained on November 11. Blood cultures grew with gram-negative rods after 3 days, which were subsequently identified as *P dispersa* with resistance to ceftazidime and susceptibility to cefepime (detailed in Table 2). The antibiotic treatment was switched to intravenous cefepime (1 g every 12 hours) at the same time because of suspected ceftazidime treatment failure and microbiological sensitivity. The fever and chill disappeared within 48 hours of the new antibiotic. On November 20, the patient remained afebrile, and the second blood culture results were negative. Chest X-ray was normal as well. In order to control the progression of the hepatic lesion, transarterial chemoembolization was performed under local anesthesia on November 23. After completion of 14 days of antibiotic treatment, the patient was discharged with no signs of recurrence.

**Table 1:** Laboratory findings of the patient's blood routine, liver function, kidney function, and electrolyte examinations.

Item	Value	Item	Value
K	4.05 mmol/L	T-Bil	18.6 μmol/L
Na	140.0 mmol/L	D-Bil	4.8 μmol/L
Cl	108.1 mmol/L	I-Bil	13.8 μmol/L
RBC	2.00 × 10^12^/L	TP	59.4 g/L
Hb	48 g/L	Alb	30.3 g/L
WBC	1.64 × 10^9^/L	GLO	29.1 g/L
Neu	1.15 × 10^9^/L	ALT	44 U/L
Plt	65 × 10^9^/L	AST	76 U/L
CRP	95.1 mg/L		

Alb = albumin, ALT = alanine aminotransferase, AST = aspartate aminotransferase, Cl = chlorine, CRP = C-reactive protein, D-Bil = direct bilirubin, GLO = globulin, Hb = hemoglobin, I-Bil = indirect bilirubin, K = potassium, Na = sodium, Neu = neutrophil, Plt = platelet, RBC = red blood cell count, T-Bil = total bilirubin, TP = total protein, WBC = white blood cell count.

**Table 2:** Antimicrobial susceptibility of *P dispersa* isolated from blood culture.

Antimicrobial agents	MIC, μg/mL	Interpretation	Antimicrobial agents	MIC, μg/mL	Interpretation
Amoxicillin	4	S	Imipenem	0.5	S
piperacillin / Tazobactam	≤4	S	Levofloxacin	≤0.12	S
Aztreonam	≤4	S	Meropenem	≤0.25	S
Ceftazidime	≤1	R	Minocycline	2	S
Cefoxitin	0.5	S	Moxifloxacin	≤0.25	S
Ciprofloxacin	16	I	Nalidixic acid	4	S
Cefpodoxime	≤0.25	S	Piperacillin	≤4	S
Colistin	2	S	Cefoperazone / Sulbactam	≤8	S
Cefotetan	≤0.5	S	Compound sulfamethoxazole	≤20	S
Cefotaxime	≤4	S	Tetracycline	2	S
Cefuroxime	≤1	S	Tigecycline	≤0.5	S
Cefuroxime axetil	16	I	Ticarcillin	16	S
Ceftizoxime	16	I	Tobramycin	≤1	S
Doxycycline	1	S	Amikacin	≤2	S
Doripenem	0.25	S	Cefepime	≤0.12	S

I = intermediary; MIC = minimum inhibitory concentration; R = resistance; S = susceptible.

### 2.7 Characteristics and preliminary identification by morphological and phenotypic profiles

**Figure 2:**
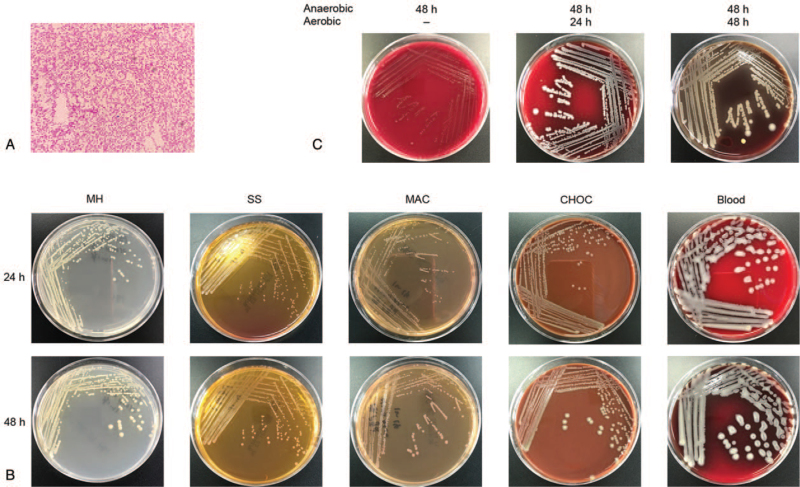
(A) Bacterial morphology after gram staining. (B) The colony morphology of bacteria in blood cultures grew on MH, SS, MAC, CHOC, and blood plates for 24 and 48 hours respectively. (C) Colony morphology and the ability to grow under aerobic and anaerobic conditions on the blood plates.

The presumptive phenotypic identification facilitated allocation of the isolates to specific VITEK 2 Test Kit for further biochemical characterization. Therefore, first of all, the selected isolates underwent presumptive phenotypic identification based on bacterial morphology after gram staining, colony morphology and the ability to grow on each media and culture conditions. The results showed that the blood cultures yielded a gram-negative bacterium rod (Fig. 2A). The culture-positive blood samples were inoculated on different plates and cultured under different conditions. The results showed that the isolated microorganism displayed yellow pigmentation, round, raised, smooth on each plate, and the colonies were facultative anaerobic (Fig. 2B and C).

The isolate was then characterized as belonging to the genus *Pantoea* with a probability of 95% by the VITEK 2 Compact automatic microbial analysis system of biochemical identification using GN cards (bioMérieux, Nürtingen, Germany). The biochemical results were shown in Table 3.

**Table 3:** Biochemical test results of vitek2 compact automatic microbial analysis system.

Item	Value	Item	Value	Item	Value
APPA	−	ADO	−	PyrA	+
IARL	−	dCEL	+	BGAL	+
H2S	−	BNAG	−	AGLTp	−
dGLU	+	GGT	+	OFF	+
BGLU	−	dMAL	−	dMAN	+
dMNE	+	BXYL	−	BAlap	−
ProA	−	LIP	−	PLE	−
TyrA	+	URE	−	dSOR	−
SAC	+	dTAG	−	dTRE	+
CIT	+	MNT	−	5KG	+
ILATk	+	AGLU	−	SUCT	+
NAGA	−	AGAL	−	PHOS	+
GlyA	−	ODC	−	LDC	−
IHISa	−	CMT	+	BGUR	−
O129R	+	GGAA	−	IMLTa	−
ELLM	−	ILATa	−		

### 2.8 Accurate identification by mass spectrometry and 16S rDNA sequencing

**Figure 3:**
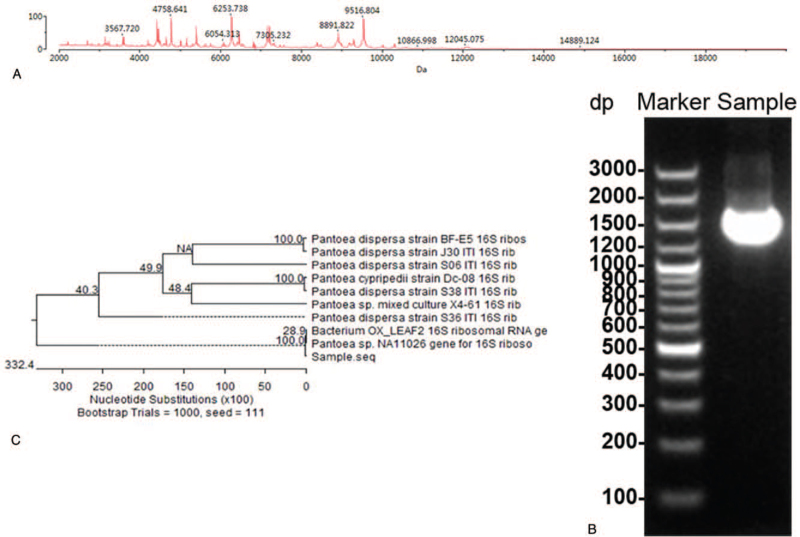
(A) MALDI-TOF MS fragmentation pattern of *P dispersa*. (B) The resulting PCR and sequencing products. (C) The 16S rRNA gene phylogenetic tree analysis of the bacterial isolate. The evolutionary history was inferred using the neighbor joining method. The isolates were aligned with closely related strains from GenBank. MALDI-TOF MS *=* matrix-assisted laser desorption/ionization time-of-flight mass spectrometry, PCR = polymerase chain reaction.

MALDI-TOF MS analysis was performed to identify the specific species of this isolate, and the result illustrated that the isolate was *P dispersa* with a probability of 99.9% (Fig. 3A). Further, to identify the bacterial isolates, approximately 1.5-kbp fragment of the 16S rRNA gene was amplified from the isolates’ genomic DNA (Fig. 3B). The pathogen was identified as *P dispersa* with 100% homology (1238 of 1478 bases) on the Ribosomal Database Project (http://rdp.cme.msu.edu/index.jsp) (Supplementary Table S3, Supplemental Digital Content). Moreover, the amplified fragments were compared with those sequences deposited in the GenBank database (Table 4). The 16S rRNA gene sequence of the bacterial isolates displayed a similarity of ≥99% to the closest known species. Based on the 16S rRNA gene, the isolate belonged to the *P dispersa* (Fig. 3C).

**Table 4:** Accession numbers of bacterial 16S rRNA gene sequences from GenBank used in the phylogenetic analysis.

Sequence description	Total score	Query cover	Identity	Accession
Pantoea dispersa strain S36 ITI 16S ribosomal RNA gene, partial sequence	2728	99%	100.00%	MT826228.1
Pantoea dispersa strain S06 ITI 16S ribosomal RNA gene, partial sequence	2728	99%	100.00%	MT826219.1
Pantoea dispersa strain J30 ITI 16S ribosomal RNA gene, partial sequence	2728	99%	100.00%	MT826213.1
Pantoea dispersa strain BF-E5 16S ribosomal RNA gene, partial sequence	2726	100%	99.93%	KY292463.1
Pantoea sp. NA11026 gene for 16S ribosomal RNA, partial sequence	2724	100%	99.93%	AB921268.1
Pantoea sp. mixed culture X4–61 16S ribosomal RNA gene, partial sequence	2724	100%	99.93%	KR029327.1
Bacterium OX_LEAF2 16S ribosomal RNA gene, partial sequence	2724	100%	99.93%	KF864672.1
Pantoea cypripedii strain Dc-08 16S ribosomal RNA gene, partial sequence	2724	100%	99.93%	KC153127.1
Pantoea dispersa strain S38 ITI 16S ribosomal RNA gene, partial sequence	2724	100%	99.93%	MT826230.1

Sequence description closely related species from GenBank database. Total score: The matching score with the closest phylogenetic relative has 0.0 E value and the number of gaps in bracket. Query cover: The percentage of the total length of your series used to calculate the score. Identity: The percentage identity with the closest phylogenetic relative of bacteria. Accession: Accession number of each sequence was provided from GenBank database.

## 3 Discussion

The genus *Pantoea*, separated in 1989, is a gram-negative, flagellated, nonencapsulated, nonspore, and capsule-forming ubiquitous brevibacterium cruentum.^[1]^ The genus *Pantoea* is generally a plant pathogen, however, to our knowledge, there is few previous disease in humans caused by this microorganism.^[5]^

The clinicians have paid more attention to the *P dispersa* isolated from the patients because of its pathogenicity.^[6]^ At present, the reported clinical cases of human infection caused by it are scarce. In 2003, a case of a 71-year-old Germany female patient with acute myeloid leukemia, multiple myeloma, and developed respiratory infection was reported. The culture of bronchoalveolar lavage of this patient grew *P dispersa*.^[3]^ In 2006, a case was reported that two adult patients developed joint infection, and the joint fluid culture revealed growth of *P dispersa*.^[7]^ In 2013, a case was reported that *P dispersa* was identified from 2 Indian neonates with early onset sepsis.^[8]^ In 2014, a case from Japan was reported that a central line-associated bloodstream infection was caused by *P dispersa*.^[9]^ In 2019, a case was reported that *P dispersa* was isolated from a patient with acute cholangitis.^[4]^ In this study, it was the first *P dispersa* identified from a patient with hepatocellular carcinoma from China.

Considering the cases mentioned above, *P dispersa* has an ability to cause infections in different systems, such as respiratory system, joint system, hematologic system, and digestive system. The patients usually develop a high fever and other symptoms caused by corresponding infected lesions. In addition, *P dispersa* has been known to cause infections not only in immunocompromised patients but also in immunocompetent patients. In this study, there is a severe immune deficit in the patient according to his history of hepatocellular carcinoma and blood routine results. Infections caused by *P dispersa* are more likely to occur in immunocompromised patients. In other words, when faced with immunocompromised patients, we cannot ignore the possibility of *P dispersa* infection. Although it seems that this pathogen is pan-sensitivity to various antibiotics as reported in cases mentioned above, this case gives us a lesson that *P dispersa* may be resistant to some antibiotics, such as ceftazidime. The clinicians need to be cautious about empirical antibiotic treatment on patients suspected of *P dispersa* infection. Therefore, accurate diagnosis of *P dispersa* infection is particularly important.

It is difficult to identify *P dispersa* accurately with conventional methods in daily work. Actually, it is VITEK 2, MS, and 16S rRNA gene sequencing that help clinicians identify *P dispersa* in this study. With traditional method, the culture characteristics of *P dispersa* are similar to *Enterobacteriaceae*. The colonies are facultative anaerobic. Phenotypic identification represents the gold standard of laboratory bacteriology, which enables us to identify 73.33% of the strains of genus or at least to family level.^[10]^ In this study, the pathogen was initially identified by biochemical tests using VITEK 2 Test Kit. The VITEK (bioMérieux Vitek, France) was an automated instrument for biochemical tests that allowed the direct inoculation of positive blood cultures into the identification cards.^[11]^ A study of this technique reported that the VITEK 2 system correctly identified 96.5% of the 113 samples on the same day that the blood culture was found to be positive when obtained directly from the blood culture bottles.^[12]^ However, the instrument can only help identify genus *Pantoea*, and the specific bacterial species still remains unknown, because the specific bacterial species is not included in the commercial database. Therefore, it is not appropriate to simply rely on biochemical tests for *P dispersa*, which may be misleading.

*P dispersa* identification needs MALDI-TOF MS analysis. MALDI-TOF MS analysis is commonly used in clinical laboratories because it can achieve species-level identification than conventional biochemical methods.^[13]^ On the one hand, its advantage is high-speed, high accuracy, and relatively low cost. On the other hand, this method may misidentify strains of the *Enterobacteriaceae* as genus *Pantoea*.^[14,15]^ For the genus *Pantoea* which is continuously revised with new species groups, the accuracy of MALDI-TOF MS analysis remains unknown.^[16]^ However, this situation may continue to improve as MALDI-TOF MS analysis for genus *Pantoea* is expanded with additional representatives of the species groups and their close relatives.

*P dispersa* identification by partial 16S ribosomal RNA (rRNA) gene sequencing is a useful diagnostic tool, with higher performance characteristics than the conventional phenotypic methods.^[17,18]^ The 16S rRNA gene is universal in bacteria largely due to its conservation across bacteria, and due to the phylogenetic signal provided by the approximately 1500 base pair (bp) locus.^[19]^ It is reported that sequence analysis of 16S rRNA identified 25 isolates in 30 isolates of rodent origin which were conventionally difficult to identify, which is more accurate than traditional methods.^[20]^ In addition, the 16S rRNA gene sequences are not restricted to a group of known bacteria and the novel isolates can be built connection to a group of related bacteria.^[21]^

In conclusion, this study discussed the characteristics of *P dispersa* and 3 different identification methods for *P dispersa*. Although *P dispersa* is rare and relatively easy to treat, clinicians still need to be vigilant, because this kind of bacteria has resistance to some antibiotics and is difficult to identify. This study will help clinicians better diagnose and treat the infection caused by *P dispersa*.

## Acknowledgments

The authors acknowledge the generous support of the Clinical Laboratory Center, Jiangsu Taizhou People's Hospital.

## Author contributions

**Conceptualization:** Yang Yang, Haitao Hu, Cheng Lin Zhou, Wenyun Zhang, Yang Yu, Qingyi Liu, Taohong Lu, Qingfang Zhang.

**Data curation:** Yang Yang, Haitao Hu, Cheng Lin Zhou, Wenyun Zhang, Yang Yu, Qingyi Liu, Taohong Lu, Qingfang Zhang.

**Formal analysis:** Yang Yang.

**Funding acquisition:** Qingfang Zhang.

**Investigation:** Yang Yang.

**Project administration:** Cheng Lin Zhou.

**Resources:** Cheng Lin Zhou.

**Supervision:** Cheng Lin Zhou, Taohong Lu, Qingfang Zhang.

**Writing – original draft:** Yang Yang, Haitao Hu, Cheng Lin Zhou, Wenyun Zhang, Yang Yu, Qingyi Liu, Taohong Lu, Qingfang Zhang.

**Writing – review & editing:** Yang Yang, Haitao Hu, Cheng Lin Zhou, Wenyun Zhang, Yang Yu, Qingyi Liu, Taohong Lu, Qingfang Zhang.

## Supplementary Material

Supplemental Digital Content

## Supplementary Material

Supplemental Digital Content

## Supplementary Material

Supplemental Digital Content
