# Supplementary material for: Characteristics and accurate identification of Pantoea dispersa with a case of spontaneous rupture of hepatocellular carcinoma in China: A case report
Source: Medicine (Baltimore). 2022 Jan 14;101(2):e28541. doi: 10.1097/MD.0000000000028541 (PMC8758028; doi:10.1097/MD.0000000000028541)
Supplement: Supplemental Digital Content [file medi-101-e28541-s001.docx]

Supplementary Table 1. Volume of PCR components designed for RNA

| Components | Volume (µl) |
| --- | --- |
| 10 X PCR Buffer |  |
| dNTP (each 10 mM) |  |
| Taq Plus DNA Polymerase（5 U/μl） |  |
| 50mM MgSO_4_ | 12.5 |
| primer F (10 µM) | 1 |
| primer R (10 µM) | 1 |
| Template (DNA) | 1 |
| ddH_2_O | 9.5 |
| Total | 25 |
