# Supplementary material for: Characteristics and accurate identification of Pantoea dispersa with a case of spontaneous rupture of hepatocellular carcinoma in China: A case report
Source: Medicine (Baltimore). 2022 Jan 14;101(2):e28541. doi: 10.1097/MD.0000000000028541 (PMC8758028; doi:10.1097/MD.0000000000028541)
Supplement: Supplemental Digital Content [file medi-101-e28541-s002.docx]

Supplementary Table 2. Parameters of thermal cycling

| Temperature（°C） | Time | Cycle |
| --- | --- | --- |
| 95 | 5 min |  |
| 94 | 30 s | 30cycle |
| 57 | 30 s |  |
| 72 | 90 s |  |
| 72 | 10min |  |
