# Supplementary material for: Characteristics and accurate identification of Pantoea dispersa with a case of spontaneous rupture of hepatocellular carcinoma in China: A case report
Source: Medicine (Baltimore). 2022 Jan 14;101(2):e28541. doi: 10.1097/MD.0000000000028541 (PMC8758028; doi:10.1097/MD.0000000000028541)
Supplement: Supplemental Digital Content [file medi-101-e28541-s003.docx]

Supplementary Table 3. Accession numbers of bacterial 16S rRNA gene sequences from Ribosomal Database Project

| Locus | Score | Identity | Sequence Description | Accession |
| --- | --- | --- | --- | --- |
| S000691510 | 1.000 | 1238 | Pantoea dispersa (T); LMG2603; | DQ504305 |
| S001332515 | 1.000 | 1155 | endophytic bacterium S03; | FJ644571 |
| S002918524 | 1.000 | 1290 | Pantoea agglomerans; RA7; | JN585671 |
| S003284039 | 1.000 | 1231 | Pantoea dispersa; R1-320; | JQ659565 |
| S003284121 | 1.000 | 1225 | Pantoea dispersa; R3-121; | JQ659647 |
| S003284127 | 0.998 | 1309 | Pantoea dispersa; R3-321; | JQ659653 |
| S003284146 | 0.999 | 1263 | Pantoea dispersa; R4-326-2; | JQ659672 |
| S003284349 | 0.997 | 1312 | Pantoea dispersa; R7-378; | JQ659875 |
| S003284413 | 0.998 | 1315 | Pantoea dispersa; R8-396; | JQ659939 |
| S003720906 | 0.999 | 1306 | bacterium Z14; | KC702727 |
| S003721870 | 0.998 | 1414 | Pantoea sp. NG9; | KC841441 |
| S003751518 | 0.997 | 1315 | Pantoea cypripedii; Dc-08; | KC153127 |
| S004091075 | 0.999 | 1372 | Pantoea sp. B2; | KJ562862 |
| S004455650 | 1.000 | 1372 | gamma proteobacterium symbiont of Plautia stali; C/Ps-TKNSm5; | LC007675 |
| S004455742 | 1.000 | 1372 | gamma proteobacterium symbiont of Plautia stali; C/Ps-ISGKm22; | LC007767 |
| S004455768 | 1.000 | 1372 | gamma proteobacterium symbiont of Plautia stali; C/Ps-ISGKf4; | LC007793 |
| S004455818 | 1.000 | 1372 | gamma proteobacterium symbiont of Plautia stali; C/Ps-YNGNf19; | LC007843 |
| S004455824 | 1.000 | 1372 | gamma proteobacterium symbiont of Axiagastus rosmarus; C/Ar-IROM; | LC007849 |
| S004455828 | 1.000 | 1372 | gamma proteobacterium symbiont of Lampromicra miyakona; C/Lm-ISGK; | LC007853 |
| S004485387 | 1.000 | 1302 | Pantoea dispersa; 1-1-b-8; | KR149609 |

Lous The locus of the closest phylogenetic. Score The matching score with the closest phylogenetic. Identity The identity with the closest phylogenetic relative of bacteria. Sequence Description Closely related species from Ribosomal Database Project. Accession Accession number of each sequence was provided from NCBI database
